# Avascular Necrosis of the Femoral Head After Palliative Radiotherapy in Metastatic Prostate Cancer: Absence of a Dose Threshold?

**DOI:** 10.7759/cureus.521

**Published:** 2016-03-06

**Authors:** Alia M Daoud, Mack Hudson, Kenneth G Magnus, Fleur Huang, Brita L Danielson, Peter Venner, Ronak Saluja, Bronwen LeGuerrier, Helene Daly, Urban Emmenegger, Alysa Fairchild

**Affiliations:** 1 Faculty of Medicine, Cross Cancer Institute, University of Alberta; 2 Oncologic Imaging, Cross Cancer Institute, University of Alberta; 3 Radiation Oncology, Cross Cancer Institute, University of Alberta; 4 Department of Oncology, Cross Cancer Institute, University of Alberta; 5 Medical Oncology, Cross Cancer Institute, University of Alberta; 6 Medical Oncology, Odette Cancer Centre, Sunnybrook Health Sciences Centre; 7 Palliative Radiation Oncology, Cross Cancer Institute, University of Alberta; 8 Radiation Therapy, Cross Cancer Institute, University of Alberta; 9 Odette Cancer Centre, Sunnybrook Health Sciences Centre

**Keywords:** avascular necrosis, palliative radiotherapy, stochastic effect

## Abstract

Avascular necrosis (AVN) is the final common pathway resulting from insufficient blood supply to bone, commonly the femoral head. There are many postulated etiologies of non-traumatic AVN, including corticosteroids, bisphosphonates, and radiotherapy (RT). However, it is unclear whether there is a dose threshold for the development of RT-induced AVN. In this case report, we describe a patient with prostate cancer metastatic to bone diagnosed with AVN after receiving single-fraction palliative RT to the left femoral head. Potential contributing factors are discussed, along with a review of other reported cases. At present, the RT dose threshold below which there is no risk for AVN is unknown, and therefore detrimental impact from the RT cannot be excluded. Given the possibility that RT-induced AVN is a stochastic effect, it is important to be aware of the possibility of this diagnosis in any patient with a painful hip who has received RT to the femoral head.

## Introduction

Avascular necrosis (AVN), also known as osteonecrosis, is caused by insufficient blood supply to the bone ultimately leading to ischemic cell death [1]. Increased osteoclastic activity attempts to remove necrotic bone, and increased osteoblastic activity occurs to repair the damage. Ultimately, the bone structures collapse; in the case of the proximal femur, this results in progressive pain exacerbated by weight-bearing and loss of joint function.

AVN most commonly affects bones with a single terminal blood supply and limited collateral circulation, such as the femoral head [2]. The postulated mechanisms of interruption of blood flow include: vascular occlusion, altered lipid metabolism, and intravascular coagulation [3]. Some of the most common causes of non-traumatic AVN of the femoral head include corticosteroids, sickle cell anemia, Gaucher’s disease, connective tissue disorders, radiotherapy, and alcohol consumption [1-4], although it may be aggravated by obesity, and can also be idiopathic [5]. Individual patients usually have more than one risk factor, indicating that the pathogenesis is likely multifactorial [6].

The exact incidence of AVN in the setting of malignancy remains unclear. Over a six year period in which pelvic x-rays were performed routinely prior to radiotherapy (RT) and yearly thereafter, 18/568 (3.2%) patients treated for gynecologic malignancies developed serious bone sequelae, with AVN diagnosed in 0.5% of patients treated for cervical cancer [7]. In that study, AVN most commonly occurred between the ages of 40 and 60 [7].

Most patients present late in the disease course. Histology is the gold standard for diagnosis but is usually unnecessary and not routinely performed. The radiographic appearance depends on the stage and extent of the lesion [8]. Early, there is an increase in density of the affected area made more prominent by surrounding osteoporosis. Cystic changes appear that represent areas of absorption of dead bone, and varying degrees of collapse are seen [8]. Recovery may be possible prior to femoral head collapse. Without treatment, the process is almost always progressive, leading to irreversible joint destruction [5].

In a prospective study in which 72 femoral heads with early AVN underwent serial magnetic resonance imaging (MRI) evaluated by blinded review, prognostic factors for the highest rates of "clinical and radiographic deterioration," were investigated. Clinical deterioration was defined as development of symptoms, while radiologic deterioration was defined as femoral head collapse. Stage at diagnosis, necrosis or more than two-thirds of the weight-bearing area, and lateral involvement (compared with medial lesions) correlated with worse outcomes [9].

RT is often cited as a risk factor for the development of AVN [3]; however, it is unclear whether the relationship is a stochastic or deterministic (non-stochastic) one. A stochastic, or probabilistic effect, has no dose threshold below which the effect does not occur, and the probability of occurrence increases with increased dose. Severity of the effect is independent of dose; the classic example is RT-induced carcinogenesis. Conversely, a deterministic effect has a practical threshold, above which the severity of harm increases with dose, as is the case with cataracts [10].

In this case report, we describe a patient with prostate cancer metastatic to bone who was diagnosed with AVN subsequent to receiving RT that included the left femoral head. This patient provided consent for his deidentified clinical information to be reported.

## Case presentation

Our patient is a 51-year-old non-smoking, non-obese (BMI 24.8) male, who worked as a surveyor, with comorbidities of hypertension, osteoarthritis, peptic ulcer disease, cataracts, left ankle surgery, migraines, depression, remote motor vehicle accident without lower extremity trauma, and minimal ethanol use (1-2 alcoholic beverages/month). He presented in 1994 with localized prostate cancer (PSA 15 ng/L; Gleason 3+4=7). He completed six months of neoadjuvant androgen deprivation therapy (cyproterone acetate) with a clinical and biochemical response, followed by radical prostatectomy with bilateral pelvic lymph node sampling confirming pT2cN0 (0/14) Gleason grade 3+4=7 adenocarcinoma. PSA nadir was 0.2.

**Figure 1:**
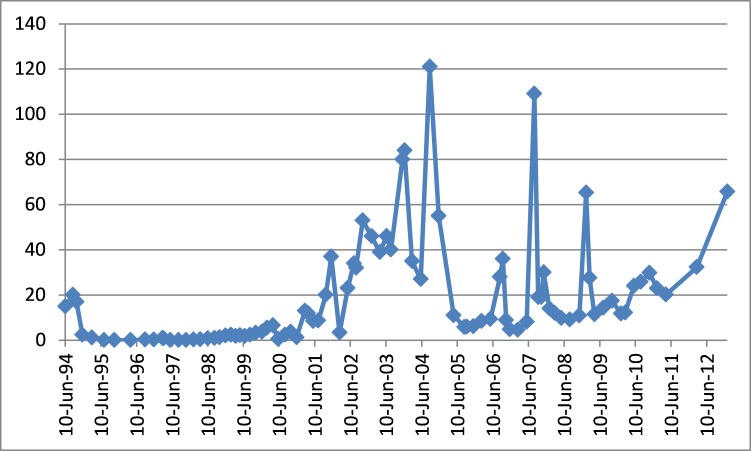
PSA values over time. Given the absolute values, it is likely that the patient’s aggressive prostate cancer was non-PSA-secreting.

By early 1997, he had experienced an asymptomatic biochemical recurrence. Restaging transrectal ultrasound (TRUS), computed tomography (CT), and bone scan were negative. A salvage prostate bed RT was recommended but declined by the patient, so he was started on diethylstilbestrol 0.1 mg od and megestrol 40 mg TID. These were discontinued within six months due to fatigue and depression in favour of watchful waiting. Further imaging between 1998 and 2001 did not reveal evidence of bone metastases despite further biochemical progression (Figure 1). During this time, he participated in a clinical trial briefly (<6 months) but was withdrawn due to non-compliance in early 2002. Ultimately, he agreed to intermittent cyproterone, acknowledging that it was not standard of care at the time.

The first diagnosis of bone metastases occurred on routine restaging in January 2004. Over the next year, he developed a significant burden of bone disease but agreed to megestrol and bicalutamide in various sequences and combinations, along with analgesics (Table 1).

**Table 1:** Morphine equivalent daily dose and steroid equivalents received. *Gabapentin started in escalating doses. **Breakthrough dose/frequency not specified so cannot be included in total MEDD. ^Diagnosed with pulmonary embolus. ¥ Received 1 dose during emergency room visit. Abbreviation: dex – dexamethasone.

Date	Estimated MEDD**	Steroids	Anti-Coagulation
July-Sept 2007	12mg	Dex 16mg IV	-
Jan 2009	22.1mg	-	-
Feb 2009	8.8mg	-	Lovenox 120mg SQ od
Feb 2009	18.9mg	-	
July 2010	12.6mg	-	
Oct 2010	37.8mg	Dex 80mg	
Dec 2010*	9.5mg	Dex 4mg po prn**	
Feb 2011	25.2mg	-	
Mar 2011	48mg	-	
Mar 2011	60mg	-	
April 2011	132mg	Dex 40mg	
May 2011	120mg	-	
Aug 2011	144mg	-	
Aug 2011	144mg	-	
Oct 2011	223.5mg	-	
Dec 2011	308mg	-	

He received a total of six months of depot luteinizing hormone-releasing hormone (LHRH) agonist with some prostate-specific antigen (PSA) and symptom control, but did not elect to continue due to side effects. Over the next two years, this patient initiated and discontinued multiple medications of his own accord, sometimes in direct contravention to his care team’s advice.

After being diagnosed with a pulmonary embolus (February 2009), he initiated self-administration of subcutaneous anticoagulation which he continued for the remainder of his life (Table 1). By October 2009, he was declared hormone-refractory but wished to remain on intermittent depot LHRH agonist only. By the spring of 2010, with progressive bone pain, he agreed to a Radiation Oncology referral.

**Figure 2:**
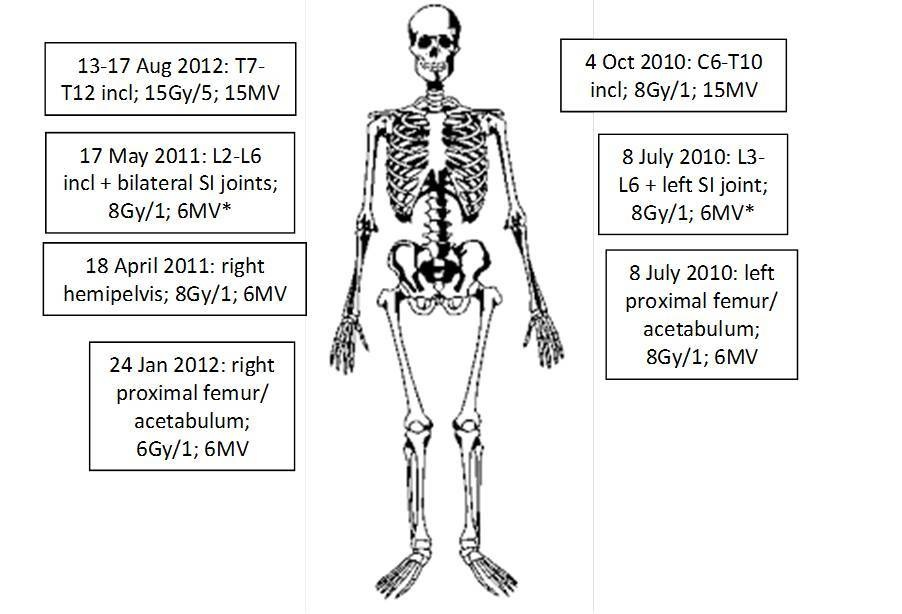
Palliative radiotherapy received. Please note, this patient has six lumbar-type vertebrae. *Femoral heads excluded. Abbreviation: incl – inclusive; SI – sacroiliac.

He would go on to receive palliative external beam RT to various anatomical locations on six different occasions (Figure 2).

**Figure 3:**
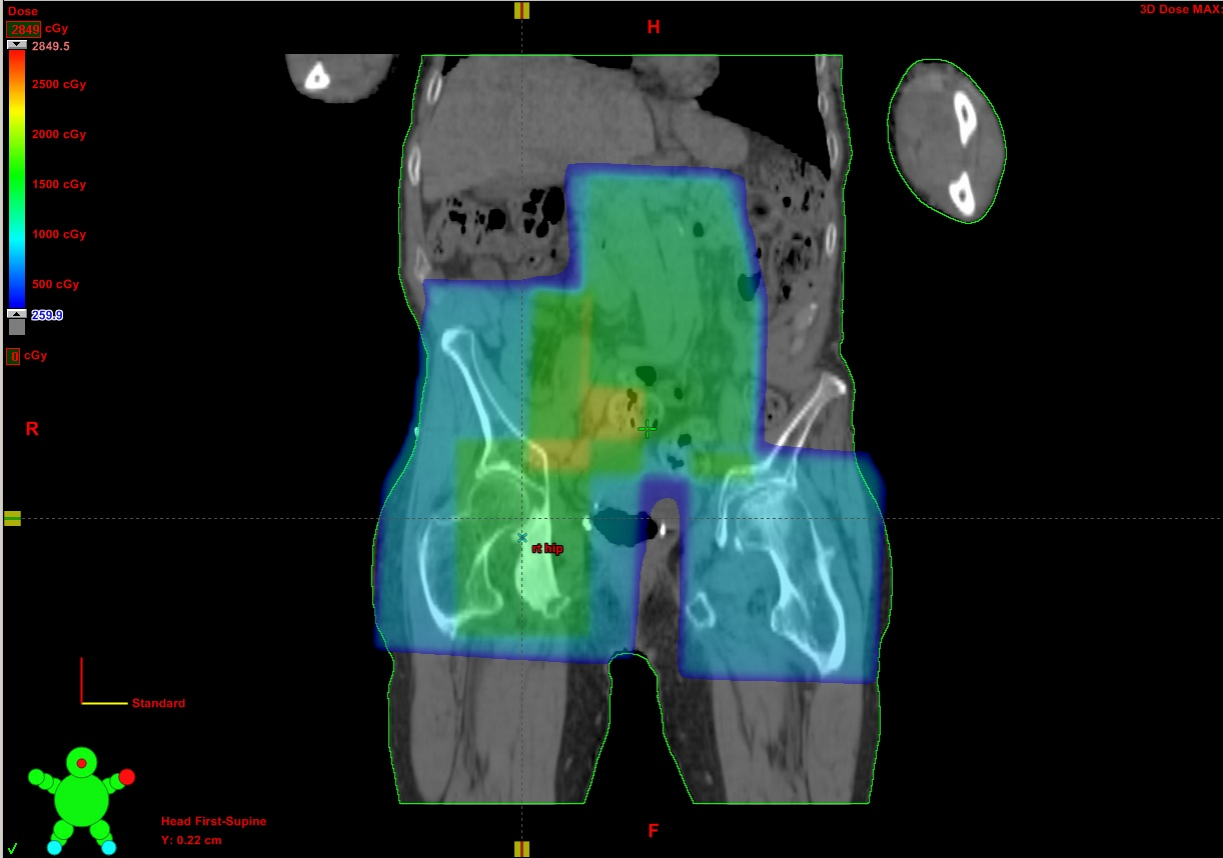
Composite dose color wash incorporating all palliative RT delivered to lumbar spine and pelvis.

He received eight Gray (Gy) in one fraction to the left hip and proximal femur via an anterior/posterior approach in July 2010 and sustained an excellent pain response. He also received 8 Gy/1 to the right hemipelvis in April 2011 and 6 Gy/1 to the right hip and proximal femur in January 2012. The lifetime doses received by the left and right femoral heads were 8.8 Gy and 15.3 Gy respectively (Figure 3).

By August 2011, with a deteriorating performance status, he was discharged from the Medical Oncology service. He went on to require multiple emergency room visits for pain control in two different provinces, and progressed to requiring a walker for ambulation. His pain was managed in the community with opioid rotation and escalation (Table 1). There is no record of him receiving bisphosphonates, vitamin D, or prednisone (for the purposes of disease control) at any time.

**Figure 4:**
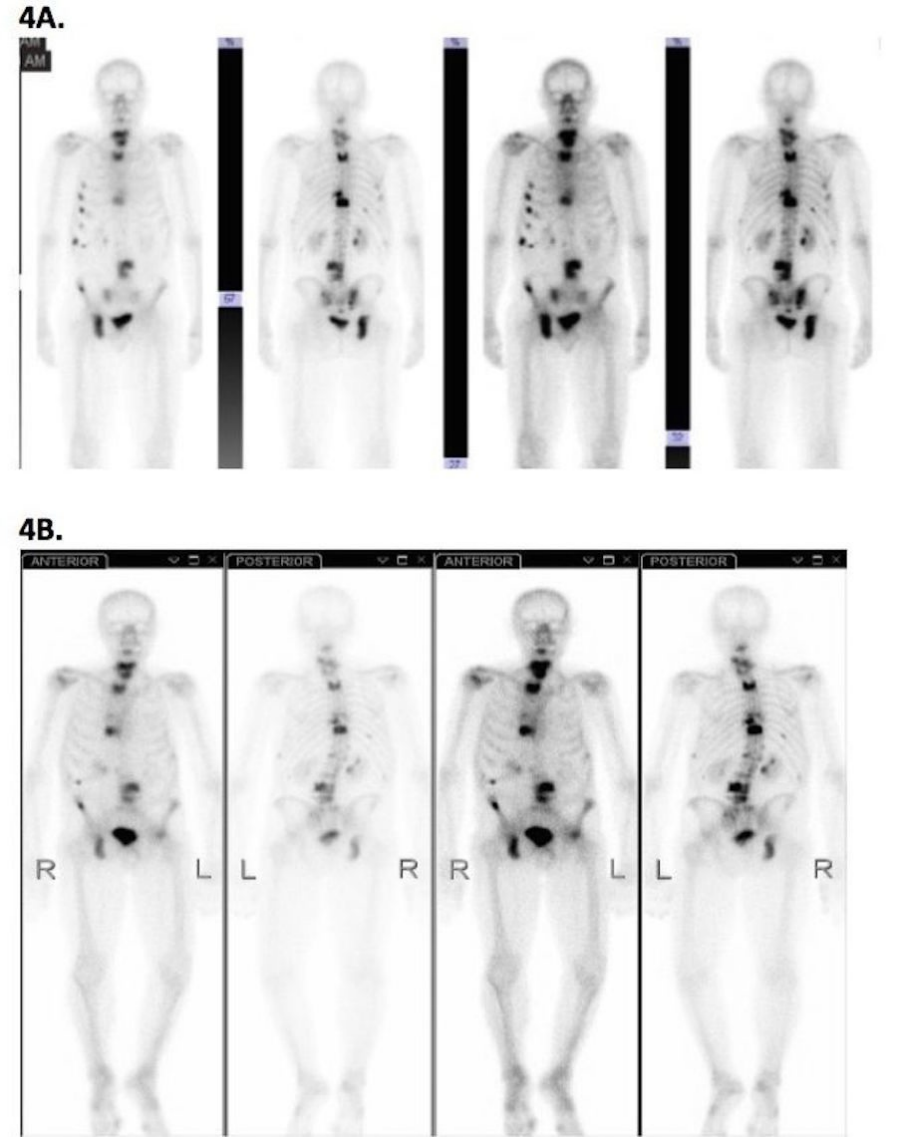
Bone scintigraphy. 4A: Bone scan performed prior to institution of RT to right pelvis showing absence of uptake on left side (Feb 23, 2011). 4B: Bone scan revealing an area of focal increased uptake in the anterior superior left femoral head in the setting of progressive bone metastases (Nov 3, 2011).

He had onset of left lateral hip pain radiating inferiorly to the knee, exacerbated by walking and other physical activity, in the fall of 2011. An abdominal flat plate performed primarily to investigate fecal loading (August 2011) had not revealed abnormalities in the left hip. A bone scan (November 2011) revealed new area of uptake in the anterior-superior left femoral head with AVN in the differential diagnosis (Figure 4).

**Figure 5:**
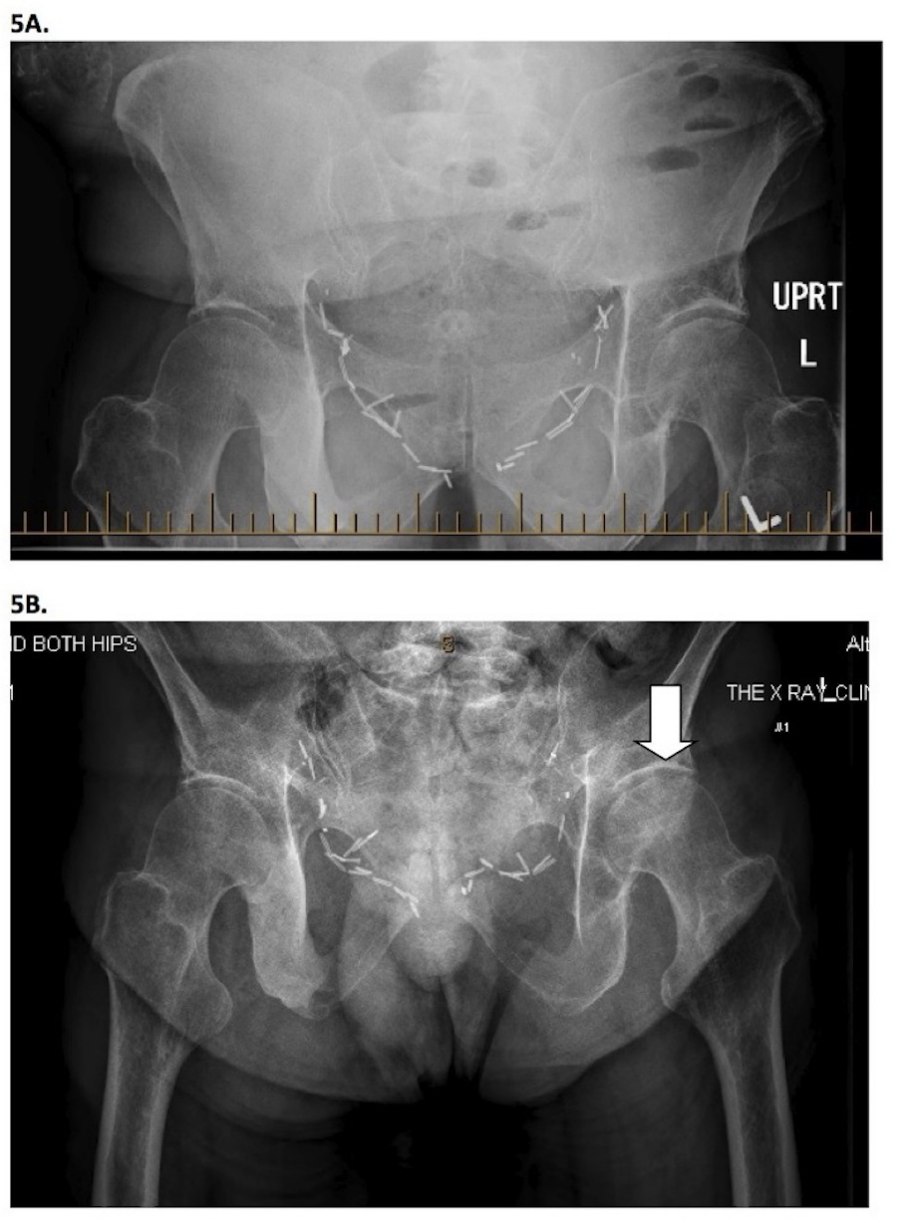
Plain x-rays. 5A: Abdominal plain film (August 11, 2011) demonstrating normal hip joints. 5B: Pelvis plain film (Nov 22, 2011) revealing flattening of the superior articular surface of the left femoral head accompanied by mild juxtaarticular sclerosis and small juxtaarticular cysts (arrow) in keeping with early avascular necrosis.

Plain films conducted three weeks later demonstrated slight flattening of the superior articular surface of the left proximal femoral head, accompanied by mild juxtaarticular sclerosis and cysts, in keeping with early AVN. In retrospect, these changes could be identified on the August imaging (Figure 5).

The diagnosis occurred approximately 15 months after RT was delivered to the left hip.

**Figure 6:**
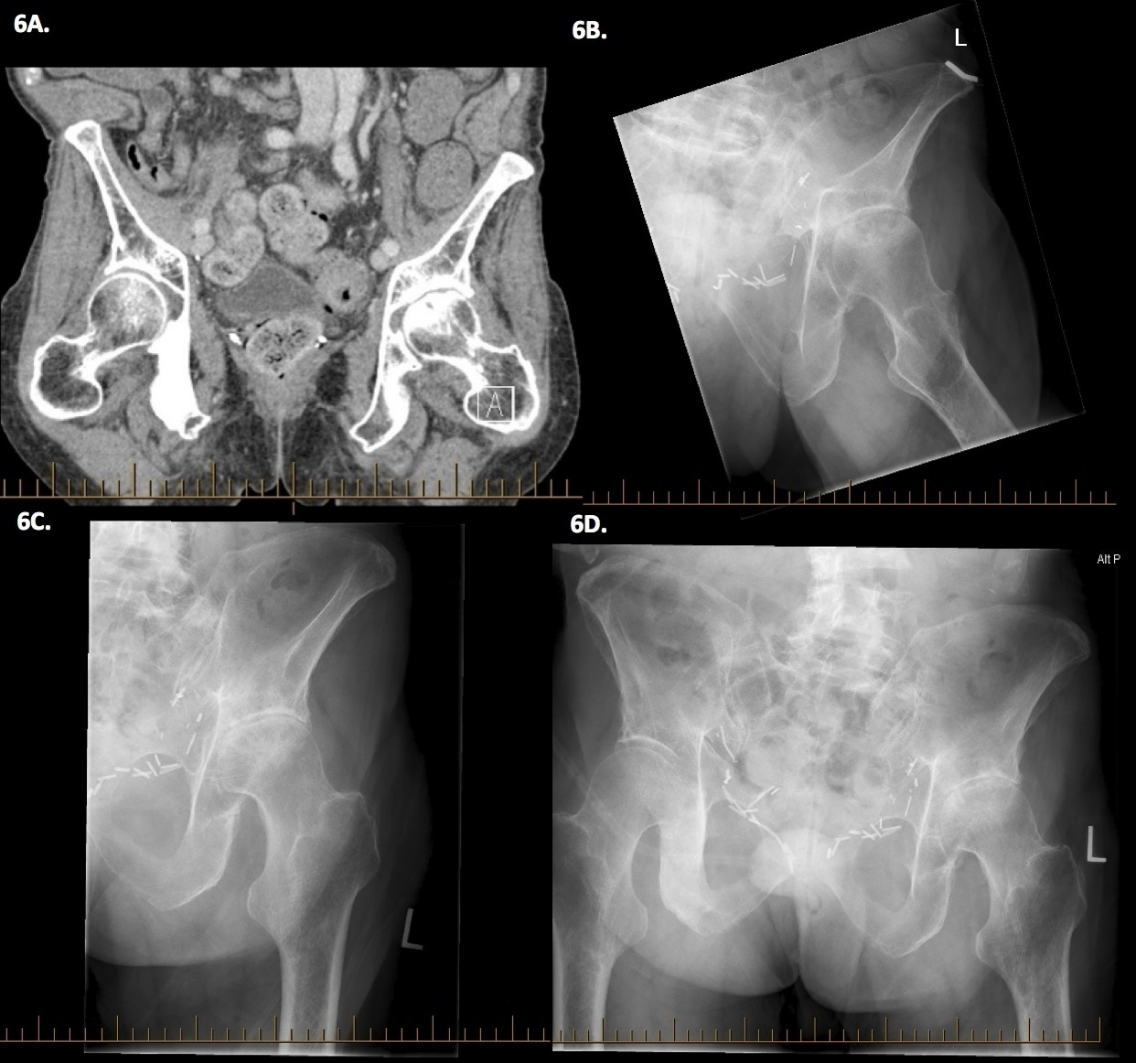
Evolution of presumed avascular necrosis. 6A: CT pelvis eight months after AVN diagnosis (July 23, 2012). The left hip demonstrates superior asymmetric joint space narrowing, with dense subarticular sclerosis and irregularity of the articular surface of the femoral head, in keeping with AVN. There is extensive dense sclerosis of the right pubic bone plus scattered sclerotic areas in both iliac bones and the left pubic bone suspicious for metastases. 6B-D: Pelvic x-rays fourteen months after AVN diagnosis (Jan 8, 2013), demonstrating severe narrowing of the joint space superiorly and laterally. Sclerosis and subchondral cyst formation is seen on both sides of the joint space. The superior articular surface is disrupted and depressed.

The patient declined further investigations including MRI. By January 2012, he was describing a constant dull ache in the left hip without neuropathic features and was walking with a pronounced limp. He described a “mushing” feeling especially when lying in a left lateral decubitus position. Two further bone scans were reported as stable in the left femoral head, with progression in his widespread bone metastases elsewhere. But CT pelvis and plain films demonstrated dramatic progression in mid 2012 and early 2013, respectively (Figure 6)​.

Taking into account his limited life expectancy due to the absence of further therapeutic options for his malignancy, the potential complications associated with surgery, and the anticipated length of post-surgical rehabilitation, hip replacement surgery was not pursued. Symptom control and supportive care were continued and the patient passed away due to his prostate cancer on April 5, 2013, approximately 17 months after the diagnosis of AVN.

## Discussion

Changes in the bone caused by radiation have been termed radiation osteitis, radiation necrosis of bone, osteonecrosis due to radiation and osteoradionecrosis [11]. Ewing ascribed the effect of RT on bone to both interference with its nutrition as a result of obliteration of the vascular supply, and to secondary irradiation related to the high calcium content [12]. Direct destruction of osteoblasts and the impairment of the regenerative response to radiation injury from compromised circulation are also likely to contribute [13]. After irradiation, the normally orderly interplay between bone formation and resorption is lost, with delayed remineralization and weakening as result [14].

We present a patient with prostate cancer metastatic to bone, who developed atraumatic AVN of the left femoral head approximately 16 months after receiving single fraction palliative-intent RT to this region. Although MRI with gadolinium contrast is considered the imaging procedure of choice, our patient declined further investigations. On balance, the diagnosis was felt to be in keeping with a predominantly AVN picture, rather than one of progressive bone metastases although we cannot absolutely exclude some contribution from the latter (Table 2). Data in Table 2 is adapted from [15]. 

**Table 2:** Differentiating metastases versus avascular necrosis. Adapted from [15].

	Metastasis	Radiation-Induced AVN
Associated soft tissue mass	Possibly	No
Type of lesion	Lytic or sclerotic in otherwise normal bone with a transition zone appreciable between normal and abnormal bone	Sclerosis without cortical destruction; fractures can occur in demineralized (as opposed to lytic) areas
Inner cortex / iliopectineal line	May be breached	Preserved
Location	Any bone via hematogenous spread	Any bone with precarious blood supply within radiation portals
Time interval	Any	Usually >1 year post-radiation

Although our patient never received systemic cytotoxic chemotherapy, bisphosphonates or other bone-modifying agents, he did receive a cumulative steroid dose equivalent to just over 9 g of dexamethasone over four years. Other potentially relevant risk factors suggested in the literature include hypercoaguability [8], although he was on therapeutic doses of anticoagulation for almost three years prior to his AVN diagnosis. An additional theory is that of osteoporosis caused by long-term androgen deprivation therapy contributing to microscopic fractures of weakened bony trabeculae, possibly exacerbated by diminished sensibility from the anti-inflammatory effects of steroids [8,16]. However, his hormonal deprivation was intermittent and incomplete, and his lifetime steroid dose relatively low. Marrow infiltration may also have played a role in weakening of the bone. Additionally, some individuals do not have blood vessels in the ligamentum teres at all, making them particularly susceptible to AVN [7]. Finally, idiopathic AVN of the femoral head has also been described [5,8], and therefore the RT may have been completely causally unrelated.

Irradiation of mature bone causes radiographically demonstrable atrophic changes, likely due to osteoblast damage, with a threshold proposed of 4000 rads (equivalent to 4000 cGy) [11]. According to currently accepted normal structure tolerance guidelines, there is a 5% risk of AVN if the entire femoral head receives 52 Gy, which rises to a risk of 50% after 65 Gy [17]. However, much smaller radiation doses—as low as 2500 rads—are sufficient to initiate changes in the endothelium of local blood vessels [18].

There is still no satisfactory explanation as to why one patient develops AVN after RT while another who has received a similar dose does not [7]. It is also difficult to explain unilateral AVN in the setting of identical doses received by the contralateral femoral head [7]. In terms of the role of RT in our case, the dose absorbed by his left femur was not considered to exceed the tissue tolerance of normal bone. In fact, it was also lower than the maximum dose received by his contralateral femoral head, which did not go on to develop AVN.

The possible explanation relates to the development of our patient’s progressively worsening bone metastases in the *right *hip, which required multiple courses of palliative RT. This led to increasing stresses being placed on the left hip as he attempted to favour the right side. As dead bone is being revascularized and remodeled, the forces involved in weight-bearing contribute to collapse and fracture [9,13]. Indeed, weight-bearing has been described as a repetitive traumatic insult, capable of exacerbating an evolving picture of AVN [2], and even repeated slight injury to tissues with a diminished capacity for recovery have been blamed for instigating AVN [5,19].

It is therefore likely that AVN occurs when a number of etiological factors coincide [6], as supported by long average latent period to the development of this complication [20]. Although the incidence of radiation-induced fracture increases after higher bone doses [7], the relationship between dose and development of AVN is not as clear-cut given the wide variety of associated total doses reported in the literature (Table 3).

**Table 3:** Biologically equivalent dose estimated from reported cases of avascular necrosis within radiated hip(s) where systemic therapy was explicitly stated as not delivered. *Maximum dose (or prescribed dose if dmax not available). **Not enough information to calculate equivalent steroid dose. Abbreviations: C – cyproterone; P – prednisone.

Reference	Histology	Hip(s) Affected	Femoral Head Dmax*	Estimated BED (Gy2)	Systemic Chemotherapy	Dexamethasone Equivalent Received Prior to Diagnosis of AVN
Present case	Prostate	Left	8.8 Gy	47.5 Gy2	No	9.1g
Macdonald patient 2 [21]	Prostate	Right	20 Gy	70 Gy2	No	C**
Thorne patient 12 [22]	HD	Right	35 Gy	65.6 Gy	No	0
Macdonald patient 1 [21]	Prostate	Bilateral	38.4 Gy	61.4 Gy2	No	C**
Phillips patient 3 [23]	Endometrial	Right	50 Gy	65.6Gy2 + 15Gy via brachy	No	0
MacDougall patient 1 [5]	Testis teratoma	Left	58.8 Gy	96.4 Gy2	No	0
Kolin patient 10 [24]	Endometrial	Left	65 Gy	125.5 Gy2	No	0
Kolin patient 11 [24]	Prostate	Right	65 Gy	125.5 Gy2	No	P**
Csuka patient 1 [25]	Prostate	Bilateral	68 Gy	128.8 Gy2	No	NR
Kolin patient 5 [24]	Breast	Bilateral	122 Gy	244 Gy2	No	60g

Other case reports have described AVN after a dose as low as 1540 rads in the absence of systemic therapy [7,26], and therefore it is likely that the total dose is not the predominant factor [2]. In the absence of being able to conclude there is a dose ceiling below which AVN does not occur, as suggested by the present case and literature review, it is therefore likely that radiation-induced AVN is a stochastic effect.

## Conclusions

In conclusion, we report a case of AVN of the femoral head after palliative RT for metastatic prostate cancer. While it is not possible to establish that radiotherapy was the dominant cause, since the etiology in this patient is likely multifactorial, the possibility of some contribution must be considered. This is especially true in the absence of significant steroid doses expected to cause osteopenia, and without cytotoxic systemic therapy or bisphosphonates. At present, the RT dose threshold below which there is no risk for AVN is unknown, and therefore involvement by the radiotherapy cannot be definitively excluded. A wide variety of radiotherapy doses delivered to the femoral head have been related to subsequent AVN diagnoses, with our patient’s dose the lowest of these.

Given the possibility that RT-induced AVN is a stochastic effect, this diagnosis should be considered in any patient with a painful hip who has received radiotherapy to the femoral head, especially in conjunction with other risk factors. Caution is required before assuming that progressive skeletal metastases are the cause, without adequate radiologic evidence excluding other etiologies. This is to avoid delivering unnecessary irradiation due to a misdiagnosis of bone metastases. An early detection will also allow for surgical intervention in eligible patients, therefore enhancing the quality of life and delaying or preventing irreversible femoral head collapse.
